# Diversifying selection in the wheat stem rust fungus acts predominantly on pathogen-associated gene families and reveals candidate effectors

**DOI:** 10.3389/fpls.2014.00372

**Published:** 2014-09-01

**Authors:** Jana Sperschneider, Hua Ying, Peter N. Dodds, Donald M. Gardiner, Narayana M. Upadhyaya, Karam B. Singh, John M. Manners, Jennifer M. Taylor

**Affiliations:** ^1^Plant Industry, Centre for Environment and Life Sciences, Commonwealth Scientific and Industrial Research OrganisationPerth, WA, Australia; ^2^Plant Industry, Black Mountain Laboratories, Commonwealth Scientific and Industrial Research OrganisationCanberra, ACT, Australia; ^3^Plant Industry, Queensland Bioscience Precinct, Commonwealth Scientific and Industrial Research OrganisationBrisbane, QLD, Australia; ^4^University of Western Australia Institute of Agriculture, University of Western AustraliaCrawley, WA, Australia

**Keywords:** rust, effector, adaptation, avirulence, selection, *Puccinia graminis*, fungal pathogens

## Abstract

Plant pathogens cause severe losses to crop plants and threaten global food production. One striking example is the wheat stem rust fungus, *Puccinia graminis* f. sp. *tritici*, which can rapidly evolve new virulent pathotypes in response to resistant host lines. Like several other filamentous fungal and oomycete plant pathogens, its genome features expanded gene families that have been implicated in host-pathogen interactions, possibly encoding effector proteins that interact directly with target host defense proteins. Previous efforts to understand virulence largely relied on the prediction of secreted, small and cysteine-rich proteins as candidate effectors and thus delivered an overwhelming number of candidates. Here, we implement an alternative analysis strategy that uses the signal of adaptive evolution as a line of evidence for effector function, combined with comparative information and expression data. We demonstrate that *in planta* up-regulated genes that are rapidly evolving are found almost exclusively in pathogen-associated gene families, affirming the impact of host-pathogen co-evolution on genome structure and the adaptive diversification of specialized gene families. In particular, we predict 42 effector candidates that are conserved only across pathogens, induced during infection and rapidly evolving. One of our top candidates has recently been shown to induce genotype-specific hypersensitive cell death in wheat. This shows that comparative genomics incorporating the evolutionary signal of adaptation is powerful for predicting effector candidates for laboratory verification. Our system can be applied to a wide range of pathogens and will give insight into host-pathogen dynamics, ultimately leading to progress in strategies for disease control.

## Introduction

The basidiomycete *Puccinia graminis* f. sp. *tritici* is the causal agent of wheat and barley stem rust. Wheat stem rust is a major threat to wheat production worldwide and hence to global food security, and has a long history of evolution to new virulent pathotypes in response to deployment of host genetic resistance (Leonard and Szabo, 2005; Pardey et al., 2013). The Ug99 strain, which first emerged in east Africa around 1999, has potential to infect most of the world's wheat (Singh et al., 2011). *Puccinia graminis* f. sp. *tritici* features a remarkably large genome of 89 Mb, which is more than four times larger than the related genome of the basidiomycete smut fungus *Ustilago maydis* (Kämper et al., 2006). Innovation of gene content has been attributed to large sets of lineage-specific expanded gene families, which are thought to drive adaptation and pathogen-associated processes (Duplessis et al., 2011; Raffaele and Kamoun, 2012).

Filamentous plant pathogens use molecules called effectors that modify host defense-related signaling, cell structure, metabolism and function to effect successful infection (Koeck et al., 2011; Giraldo and Valent, 2013). These pathogens deliver effector molecules either to the host apoplast or translocate them directly into the host cytoplasm, often through specialized infection structures such as haustoria. Plants defend themselves against pathogen attacks by using surface and intracellular recognition mechanisms, part of which directly recognize effectors and trigger resistance reactions (Dodds and Rathjen, 2010). Over time these interactions lead to an evolutionary arms race between host and pathogen (Anderson et al., 2010). Therefore, effectors are expected to be amongst the most rapidly evolving genes in pathogen genomes in a recurring strategy to circumvent plant resistance.

Advances in next-generation sequencing technologies are yielding a growing number of sequenced pathogen genomes, which allow a comprehensive and objective study of the evolutionary arms race between host and pathogen through the analysis of large-scale divergence genomic data. Genes undergoing purifying selection evolve slowly to maintain their conserved function. Weakened purifying selection occurs when a gene can mutate freely and randomly with little penalty because it has little or restricted functional significance. On the other hand, positive natural selection occurs when changes with functional consequences are favored as it produces high variability and adaptability. This is often observed in pathogenicity-related genes of microbes, which must adapt to the changing host environment and avoid immune recognition. The relative rates of synonymous (*d_S_*) and non-synonymous substitutions (*d_N_*) in genes is commonly used to assess selection. A *d_N_*/*d_S_* ratio <1 indicates purifying selection and functional conservation, *d_N_*/*d_S_* = 1 is consistent with neutral evolution, and *d_N_*/*d_S_* > 1 is indicative of diversifying selection or potential functional divergence.

A diverse range of methods for estimating diversifying selection (counting methods, pairwise *d_N_*/*d_S_* ratios, maximum likelihood codon specific estimates) are available (Aguileta et al., 2009). Calculation of a global *d_N_*/*d_S_* ratio is relatively simple, but has low sensitivity since in many cases diversifying selection acts only on certain sites of the protein domains. Computational diversifying selection analysis estimated by maximum likelihood is a more sophisticated procedure to test for selection pressure on individual codons that can also take into account the lineage of genes within a family (Yang and Nielsen, 2000, 2002). These methods are powerful but they strongly depend on the quality of the multiple sequence alignment, the phylogenetic tree, the level of sequence divergence and the sample size. The software package PAML (Yang, 1997, 2007) implements sophisticated methods for estimating the *d_N_*/*d_S_* ratio and aims to detect positively selected sites in a number of genes by using varying *d_N_*/*d_S_* ratios among sites and Bayes Empirical Bayes (BEB) analysis (Yang et al., 2005). Sites with posterior probability of greater than 95% (or 99%) are inferred as positively selected codons.

Signatures of diversifying selection have been predicted computationally in several filamentous plant pathogen effectors (Ma and Guttman, 2008; Aguileta et al., 2009). In the highly polymorphic phytotoxin-like *scr74* gene family of the oomycete *Phytophthora infestans*, evidence of diversifying selection in the mature protein region was found (Liu et al., 2005). Diversifying selection was also detected in RXLR effector paralogs, acting on the C-terminal regions of the proteins (Win et al., 2007). In the fungal host-specific necrotrophic effector *ToxA*, produced by the wheat pathogens *Pyrenophora tritici-repentis* and *Parastagonospora nodorum*, two codons were predicted to be under diversifying selection using polymorphism data (Stukenbrock and McDonald, 2007). Likewise, four codons were predicted to be under diversifying selection in the necrotrophic effector *Tox1*, produced by *Parastagonospora nodorum* (Liu et al., 2012). Six genes encoding cell wall degrading enzymes in *Zymoseptoria tritici* were also found to undergo diversifying selection in either host adaptation or host evasion processes (Brunner et al., 2013). Plant resistance (*R*) genes control recognition of pathogens carrying specific avirulence (*Avr*) effectors in a gene-for-gene model. For several avirulence proteins, signatures of diversifying selection have been detected. For instance, the *AvrL567, AvrP123*, and *AvrP4* genes are highly polymorphic in *Melampsora lini* and show evidence of diversifying selection consistent with an evolutionary arms race (Dodds et al., 2006; Barrett et al., 2009). Signatures of diversifying selection in *Phytophthora* effectors have also been linked to the ability to jump to another host (Dong et al., 2014).

The prediction of pathogenicity-associated proteins such as fungal effectors is an ongoing challenge. The most common technique is to return a set of proteins that have a predicted secretion signal and additionally are small and cysteine-rich, despite increasing awareness in the literature that not all effectors share these features (Ellis et al., 2009; Sperschneider et al., 2013). For example, the flax rust avirulence effectors AvrL567 and AvrM are devoid of cysteines (Dodds et al., 2004; Catanzariti et al., 2006). Furthermore, the number of effector candidates will be overwhelmingly large, making target selection for functional testing in the laboratory difficult. For example, Duplessis et al. (2011) report 1106 proteins in *P. graminis* f. sp. *tritici* that fit the following criteria as candidate effectors: a predicted secretion signal, no transmembrane region, no GPI-anchor site and size smaller than 300 amino acids. These candidate effectors were predicted to form 164 clusters, varying in membership from 2 to 44, and form a striking 10% of all of the expanded families in the stem rust genome. Another bioinformatic pipeline uses Markov and hierarchical clustering to identify protein families of wheat stem rust and poplar leaf rust, and it prioritizes effector candidates using a ranking system based on eight criteria associated with effector properties (Saunders et al., 2012). In both approaches, selection of gene families as candidate effectors strongly depends on the accuracy of the secretion prediction tools and the validity of the thresholds used for small size and cysteine-rich.

Instead of making *a priori* assumptions on effector candidate properties solely on the sequence level, in this work we combine three layers of evidence (taxonomic information, *in planta* up-regulation, diversifying selection) to predict effector candidates in a pathogen genome that is highly capable of host adaptation. First, we use comparative information from publicly available fungal genomes to separate the predicted gene families into those that are specific to pathogenic fungi and into those that are found across pathogenic and non-pathogenic fungi. Both pathogen-associated and fungal gene families are then grouped using unsupervised clustering based on a broad set of 35 sequence-derived protein features to find putative effector protein clusters (Sperschneider et al., 2013). If small, cysteine-rich proteins with a secretion signal are a class of proteins that are distinct from the remaining proteins, they can be expected to show up as a cluster with enrichment in those features. To add further evidence for a protein's involvement in pathogenicity, expression data and signatures of diversifying selection are incorporated to prioritize effector candidates. A combination of these three layers of evidence leads to a small set of effector candidates that have a likely role of interacting with the host plant and are well supported candidates for future laboratory testing.

## Materials and methods

### Prediction of pathogen-associated and fungal gene families

For the prediction of gene families, we used Tribe-MCL (Enright et al., 2002) with all-vs.-all phmmer bit scores (Finn et al., 2011) on the *P*. *graminis* f. sp. *tritici* protein set. Both Tribe-MCL and phmmer were run with default parameters. For each member in a gene family, we recorded the phmmer hit distribution to 72 publicly available fungal genomes from the JGI MycoCosm (Grigoriev et al., 2012), supplemented by five genomes of *Fusarium pseudograminearum* and the two genomes *Fusarium acuminatum* and *Fusarium incarnatum—F. equiseti* (Gardiner et al., 2012; Moolhuijzen et al., 2013). This resulted in a set of 78 fungal genomes which includes 24 non-pathogens (saprophytes, non-pathogenic yeasts) and 54 pathogens (pathogens, parasites, ectomycorrhizal, symbionts, mycoparasitic) (see Table S1 for genome list and classification). For a given protein *x_i_* with its corresponding list of phmmer hits that cover at least 60% of query and target sequence, pathogen precision is calculated as follows:

$$P\left(i\right)=100\times \frac{\#\text{hits to fungal pathogens}}{\#\text{ hits}}$$

For a gene family, *P(i)* is calculated by taking the average pathogen precision over its protein members. Gene families with *P(i)* > 90% were classified as pathogen-associated.

### Prediction of putative effector clusters and expression data

For each pathogen-associated or fungal gene family, a representative member was chosen and a previously developed protein *k*-means clustering method was applied to the pathogen-associated and fungal gene family representatives to find putative effector protein clusters across the gene families (Sperschneider et al., 2013). Each representative gene family member was assigned a 35-dimensional feature vector based on the average values for the corresponding gene family members. The 35-dimensional feature vector contains the protein length, the *D*-score returned by SIGNALP 4.1 (Petersen et al., 2011), the score for extracellular localization site prediction calculated by WoLF PSORT (Horton et al., 2007), the molecular weight, protein charge, isoelectric point, amino acid composition as well as the classification of amino acid composition (tiny, small, aliphatic, aromatic, non-polar, polar, charged, basic, and acidic). The features were calculated using pepstats from the EMBOSS package (Rice et al., 2000). All of these 35 features were calculated for each gene family member and the average values across the whole gene family were assigned as the feature vector for the gene family representative. The elbow plot method was used to estimate the number of clusters for the *k*-means clustering method, both using SciPy. After clustering, Mann–Whitney *U*-tests using R were conducted for each feature in the 35-dimensional vector to test whether the distribution within a cluster is identical to the full background distribution, i.e. all clusters. Highly significant *p*-values for both directions (lesser and greater, *p* < 2.2 × 10^−16^) were recorded. Putative effector clusters were chosen based on elevated levels of expression during infection using stem rust expression data. Stem rust expression data from Duplessis et al. (2011) was accessed at the GEO database (http://www.ncbi.nlm.nih.gov/geo/) as series GSE25020 and the tool GEO2R was used to identify differentially expressed stem rust genes by comparing *in planta* wheat infection to germinated urediniospores. Genes with fold change of at least two were reported as up-regulated during infection. *P*-values were adjusted using the Benjamini & Hochberg method, genes with *p* < 0.05 were reported as significant and genes with *p* < 0.00001 were labeled as highly significant. RNAseq expression data of isolated haustoria was compared to that of germinated urediniospores of wheat stem rust strain 21–0 and transcripts with normalized fold changes >2 were reported as haustorial up-regulated genes (Upadhyaya et al., in press).

### Diversifying selection analysis

The analysis of diversifying selection was performed for the Pucciniomycotina branch of the fungal kingdom, which includes the following genomes:

- *Puccinia graminis* f. sp. *tritici* (Duplessis et al., 2011).
- *P. triticina* 1-1 BBBD Race 1 [*Puccinia* Group Sequencing Project, Broad Institute of Harvard and MIT (http://www.broadinstitute.org/)].
- *P. striiformis* PST-78 [*Puccinia* Group Sequencing Project, Broad Institute of Harvard and MIT (http://www.broadinstitute.org/)].
- *Melampsora lini* (Nemri et al., 2014).
- Melampsora laricis-populina (Duplessis et al., 2011).
- *Sporobolomyces roseus* [unpublished data, with permission from the JGI MycoCosm (Grigoriev et al., 2012)].

For each protein in *P. graminis* f. sp. *tritici*, phmmer (Finn et al., 2011) was run against the Pucciniomycotina genomes, and all significant protein hits (*E*-value < 10^−5^) and per-domain hits per protein were recorded. Significant protein hits were kept if the combined domain hits for query and target cover more than 60% of the sequences, respectively. This ensured that for the subsequent diversifying selection analysis only reliable and well-conserved multiple alignments were used. Protein multiple sequence alignments were inferred using PRANK with the +F option (Loytynoja and Goldman, 2005), which has been shown to outperform other alignment methods in diversifying selection analyses (Fletcher and Yang, 2010). Alignment columns with more than 70% gap characters were masked using custom Python scripts for preparation of phylogenetic tree prediction. Phylogenetic trees were calculated using the Phyml package version 20120412 (Guindon et al., 2010). Trees were midpoint rooted and orthologs were derived with the species overlap method using ETE (Huerta-Cepas et al., 2010). Each ortholog set was aligned, gaps were masked and phylogenetic trees were predicted as described above. The gaps in the ortholog protein alignments were used to produce a coding sequence alignment using Pycogent (Knight et al., 2007) for input to PAML. Site-specific diversifying selection on ortholog sets was calculated using PAML, with alignment gaps removed. Two likelihood ratio tests of site-specific diversifying selection were used: model M1 (neutral) to model M2 (selection) and model M7 (beta) to M8 (beta&ω) and *P*-values < 0.05 were reported as significant.

## Results

### A pipeline for predicting effector candidates in expanded pathogen genomes using multiple lines of evidence

The wheat stem rust *Puccinia graminis* f. sp. *tritici* has shown the ability to rapidly overcome previously resistant wheat cultivars and, like many other filamentous plant pathogens, features a large genome with expanded gene families that have been linked to host-pathogen interactions (Duplessis et al., 2011; Raffaele and Kamoun, 2012). Adaptive selection and genetic variation are the driving forces behind the evolutionary arms race between host and pathogen, and effector proteins can be expected to be amongst the most rapidly evolving genes in pathogen genomes. Existing effector prediction pipelines that focus on the set of secreted, small, cysteine-rich proteins are prone to return an overwhelming number of candidate effectors and furthermore, not all true effectors will fit these criteria. In the following, we show that a small set of strong effector candidates can be predicted by combining multiple lines of evidence (pathogen-specific, highly expressed during infection, rapidly evolving), without making *a priori* assumptions about their protein properties.

**Figure 1:**
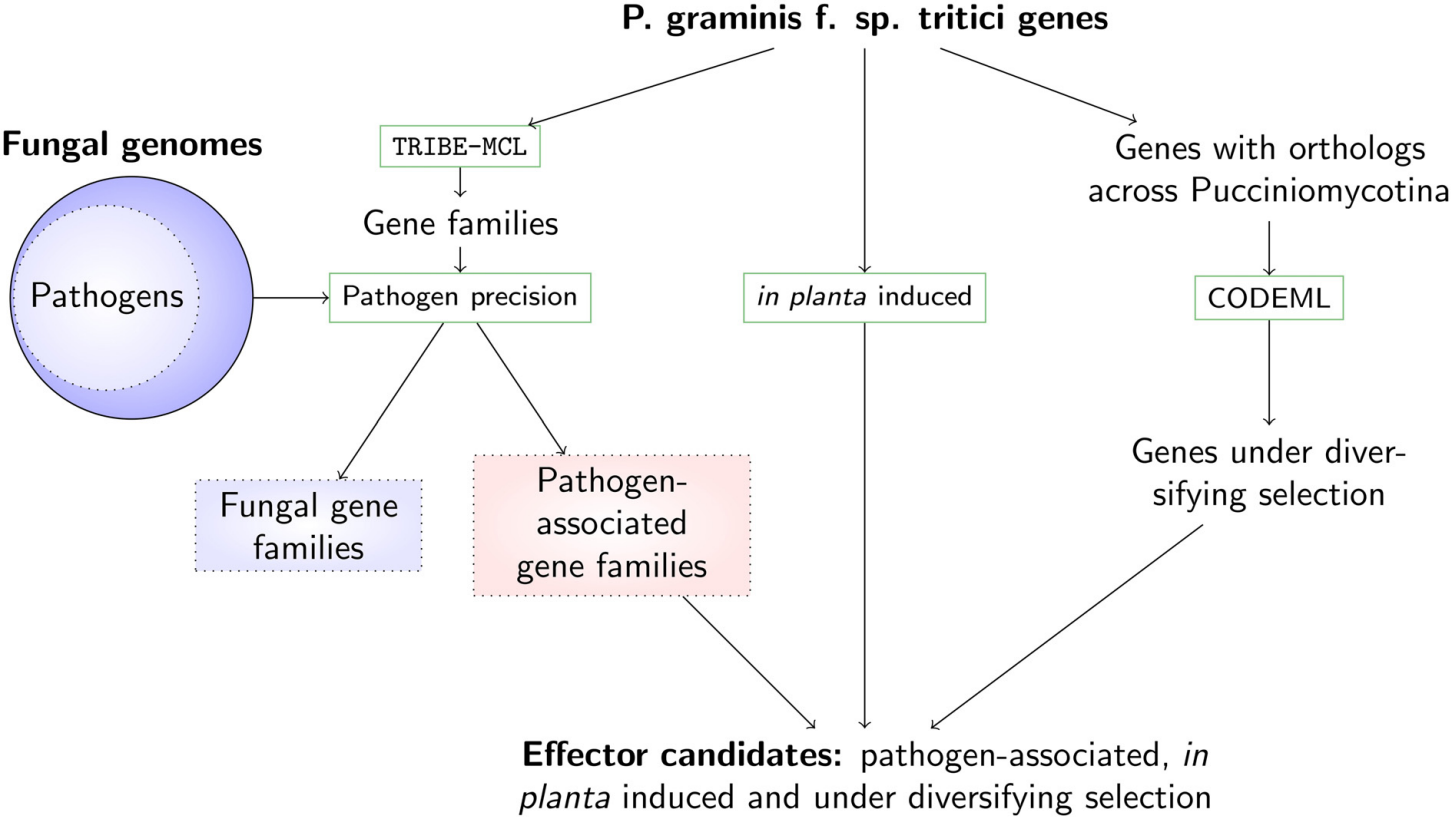
**A pipeline for predicting effector candidates in expanded pathogen genomes incorporating comparative analysis, expression data and evolutionary support**. The stem rust genes are grouped into gene families using TRIBE-MCL (Enright et al., 2002) and then split into families associated with pathogenicity (including those that are specific to *P. graminis* f. sp. *tritici*) and into fungal families, using comparative information from 78 fungal genomes. To limit the number of putative effector candidates, we use two additional lines of evidence: up-regulation during *in planta* infection and signatures of diversifying selection. Genes that are rapidly evolving, induced during infection and conserved predominantly across pathogenic fungi are returned as prime effector candidates.

We prioritize effector candidates in the stem rust *P. graminis* f. sp. *tritici* by linking comparative data, patterns of diversifying selection, and expression data during infection to a gene family's likelihood of participation in host-pathogen interactions (Figure 1). Using comparative information from 78 fungal genomes (pathogens and non-pathogens, Table S1), gene families were divided into those that can be associated with pathogenicity and those that are not related to pathogenicity (with the latter referred to as fungal genes). To dissect the nature of the gene families on a protein similarity level, a gene family representative was randomly chosen and the averages of 35 sequence-derived features for the gene family (e.g., average signal peptide prediction score, average molecular weight, average amino acid composition, see Materials and Methods) were assigned to each representative. This analysis then allowed us to use a previously developed unsupervised *k*-means clustering technique of sequence-derived features (Sperschneider et al., 2013). Enrichment and depletion analysis of the predicted protein clusters could then show whether putative effector clusters with certain characteristic features (e.g. secretion signals, small amino acids and cysteines) were present in an unbiased way, and whether commonalities or differences across pathogen-associated and fungal gene families existed. To include further evidence for pathogenicity function, ortholog sets were analyzed using prediction of their likelihood of undergoing diversifying selection. Pairwise *d_N_*/*d_S_* ratios from entire genes can only indicate diversifying selection if it has acted on all or the majority of the codons, which is rarely the case. Diversifying selection prediction is more powerful using site-specific or branch-specific models, which allow varying *d_N_*/*d_S_* ratios across codons or lineages. Proteins that were up-regulated *in planta* and were observed as part of pathogen-associated gene families predicted to undergo diversifying selection were selected as strong effector candidates for *P. graminis* f. sp. *tritici*.

### Pathogen-associated and fungal gene families form distinct protein clusters

Lineage-specific expanded gene families in *P. graminis* f. sp. *tritici* are thought to drive genome innovation, adaptation and pathogen-associated processes. To investigate gene families that can be associated with pathogenicity, the set of 15,979 *P. graminis* f. sp. *tritici* genes was first collapsed into gene families using Tribe-MCL with all-vs.-all phmmer bit scores (Enright et al., 2002; Finn et al., 2011). This resulted in 2351 predicted gene families covering a total of 15,387 genes with an additional 592 single genes. The gene families were then examined regarding their conservation across the fungal kingdom using 78 publicly available fungal genomes (Materials and Methods). The 78 fungal genomes were classified as either non-pathogenic (saprophytes, non-pathogenic yeasts) or pathogenic (pathogens and plant-associated fungi such as parasites, ectomycorrhizal, symbionts, mycoparasitic). This method suggests 1210 pathogen-associated gene families (including those that are specific to *P. graminis* f. sp. *tritici*) and 1141 fungal gene families not associated with pathogenicity.

An ongoing question is whether fungal effector proteins share characteristics on the sequence or structure level. To begin to address this question, an existing *k*-means clustering technique based on 35 sequence-derived protein features was applied (Sperschneider et al., 2013). Briefly, each protein has 35 features ranging from signal peptide prediction score to amino acid composition. Unsupervised *k*-means clustering is used to predict protein groups that share features distinctive from other groups. From this, enrichment or depletion in characteristic features was calculated for each cluster. However, expanded gene families in the stem rust genome are expected to share a high degree of sequence similarity and a clustering step based on sequence-derived features is likely to group existing gene families together instead of looking for functional commonalities between gene families potentially involved in pathogenicity. Therefore, a representative member was chosen for each gene family and was assigned the average 35 sequence-derived feature vector for the corresponding gene family. This methodology avoids clustering according to sequence similarity within the gene families.

For the 1210 pathogen-associated gene families, the *k*-means clustering returns 12 protein clusters, whereas the 1141 fungal gene families are predicted to form 10 clusters (Table 1). Enrichment and depletion analysis shows the characteristic features of each cluster and reveals two clusters of secreted proteins, one across the pathogen-associated gene families and one across the fungal gene families (Table 1, C6 and C7). The pathogen-associated secreted cluster C6 is enriched in tiny and non-polar amino acids as well as cysteines and glycines. The enrichment in cysteines and small size are features that are commonly associated with fungal effector proteins. Interestingly, only 66.5% of proteins in cluster C6 are predicted to contain a signal peptide by SignalP 4.1 (Petersen et al., 2011), suggesting that they may be clustered according to unifying features that go beyond the signal peptide prediction score. The fungal gene families also contain a cluster with enrichment in secretion signals and tiny and small amino acids as well as glycines (Table 1, C7), however without enrichment in cysteines.

**Table 1:** **Properties for the clusters of pathogen-associated and fungal gene families are shown**.

**Cluster**	**# of gene families**	**# of proteins**	**Enrichment** ↑	**Depletion** ↓
**PATHOGEN-ASSOCIATED GENE FAMILIES**				
C1	131	1012	–	Protein charge, basic
C2	88	751	Polar, charged, acidic, D, E	Protein charge
C3	53	324	Protein charge, basic, K	–
C4	72	342	Molecular weight	–
C5	110	533	R	Molecular weight, acidic, N, D
C6	117	871	Secretion, extracellular, tiny, non-polar, C, G	Molecular weight, charged, acidic, R, E
C7	155	1033	Polar, S	Aliphatic, V
C8	70	389	Tiny, small	Aromatic, charged, basic
C9	73	307	Aliphatic, non-polar, I	Charged, acidic, D
C10	18	112	–	–
C11	153	1709	Aromatic, charged, acidic, E, I, K, F	Tiny, small, A, P, S, T
C12	170	1468	–	–
**FUNGAL GENE FAMILIES**				
C1	131	1045	–	Tiny, small, P, S
C2	70	416	Molecular weight	–
C3	183	1113	Aromatic, H, W	–
C4	151	1031	Small, polar, P, S	Aliphatic, I, V
C5	103	493	Polar, charged, basic, D, E, K	Aliphatic, aromatic
C6	135	683	–	Protein charge
C7	73	451	Secretion, extracellular, tiny, small, G	Charged, basic, R, E
C8	135	573	Aliphatic, non-polar, A, G, V	–
C9	100	513	Aliphatic, aromatic, non-polar, I, L, F	Charged, basic, acidic, D, E, Q, K
C10	60	218	Protein charge, charged, basic, R, K	–

*For each characteristic in the 35-dimensional feature vector, Mann–Whitney U-tests were used to test whether the distribution within a cluster is identical to the full background distribution for all clusters and highly significant p-values for both directions (lesser ↓ and greater ↑) are shown. Secretion refers to the predicted Signal P score and extracellular score to the WoLF PSORT score. The following amino acid memberships are used: tiny (A, C, G, S, T), small (A, C, D, G, N, P, S, T, V), aliphatic (A, I, L, V), aromatic (F, H, W, Y), polar (D, E, H, K, N, Q, R, S, T), charged (D, E, H, K, R), basic (H, K, R), and acidic (D, E)*.

We searched the Pfam database for significant hits (E-value < 10^−5^) to all proteins in the secreted clusters. The pathogen-associated secreted cluster C6 has significant Pfam domain hits only for 53 of its 871 proteins (6.1%), which is expected as the vast majority of fungal effector proteins are known to lack functional annotation. On the other hand, the fungal secreted cluster C7 could be confidently annotated with Pfam domain hits for 327 of its 451 proteins (72.5%). Table 2 shows the most frequent Pfam domain hits for the two clusters. The fungal secreted cluster C7 contains a large number of proteins involved in phospholipase activity (PF01735), glycosyl hydrolase activity (PF00704 and PF00150), proteolysis (PF00026) and superoxide dismutase activity (PF00080). The pathogen-associated secreted protein cluster C6 only has a few Pfam domain hits, which does not allow for functional annotation.

**Table 2:** **The most frequent Pfam domain hits are shown for the two clusters with an enriched secretion signal across the pathogen-associated and fungal gene families**.

**Cluster**	**Pfam domains**	**Pfam ID**	**# of proteins**	**InterPro description**
**PATHOGEN-ASSOCIATED C6**				
	Triglyceride lipase	PF01764	4	Triglyceride lipases are lipolytic enzymes that hydrolyse ester linkages of triglycerides
	Copper/zinc superoxide dismutase	PF00080	3	Metalloproteins that prevent damage by oxygen-mediated free radicals
	Dioxygenase	PF00775	2	Dioxygenases catalyze the incorporation of both atoms of molecular oxygen into substrates using a variety of reaction mechanisms
	Glycosyl hydrolases family 43	PF04616	2	Widespread group of enzymes that hydrolyse the glycosidic bond between two or more carbohydrates, or between a carbohydrate and a non-carbohydrate moiety
**FUNGAL C7**				
	Lysophospholipase	PF01735	18	Phospholipase activity
	Glycosyl hydrolases family 18	PF00704	17	Widespread group of enzymes that hydrolyse the glycosidic bond between two or more carbohydrates, or between a carbohydrate and a non-carbohydrate moiety
	Cellulase (glycosyl hydrolase family 5)	PF00150	16	Degradation of cellulose and xylans
	Eukaryotic aspartyl protease	PF00026	16	Aspartic-type endopeptidase activity, proteolysis
	Copper/zinc superoxide dismutase	PF00080	16	Metalloproteins that prevent damage by oxygen-mediated free radicals

*For the two clusters, a Pfam search was performed for all members. The proteins in the pathogen-associated cluster C6 predominantly lack functional annotation, whereas the fungal secreted cluster C7 has Pfam domain hits for the majority of its proteins. For each cluster, the top five Pfam domain hits are shown if they have at least two members*.

Taken together, this analysis suggests that there are distinct groups of secreted proteins that can be separated by means of comparative genomics. We predicted a cluster of fungal gene families with an enriched secretion signal that contains proteins with putative enzymatic roles related to plant biopolymers. In contrast, across the pathogen-associated gene families, we predicted a cluster with enrichment in secretion signals, tiny and non-polar amino acids as well as cysteines and glycines. The vast majority of these proteins lack functional annotation. This further supports the hypothesis that secreted, small, cysteine-rich proteins play a role specifically in the pathogenicity of *P. graminis* f. sp. *tritici*.

### Small, cysteine-rich proteins in pathogen-associated gene families show elevated levels of up-regulation during infection

To investigate whether differences in expression levels can be found between fungal and pathogen-associated gene families, the number of up-regulated genes was calculated for each cluster given in Table 1. We used expression data from Duplessis et al. (2011) to identify differentially expressed stem rust genes by comparing *in planta* wheat infection to germinated urediniospores. Genes which have differential expression with *p* < 0.05 are reported as significant and those with *p* < 0.00001 as highly significant, both requiring a fold change >2.

**Figure 2:**
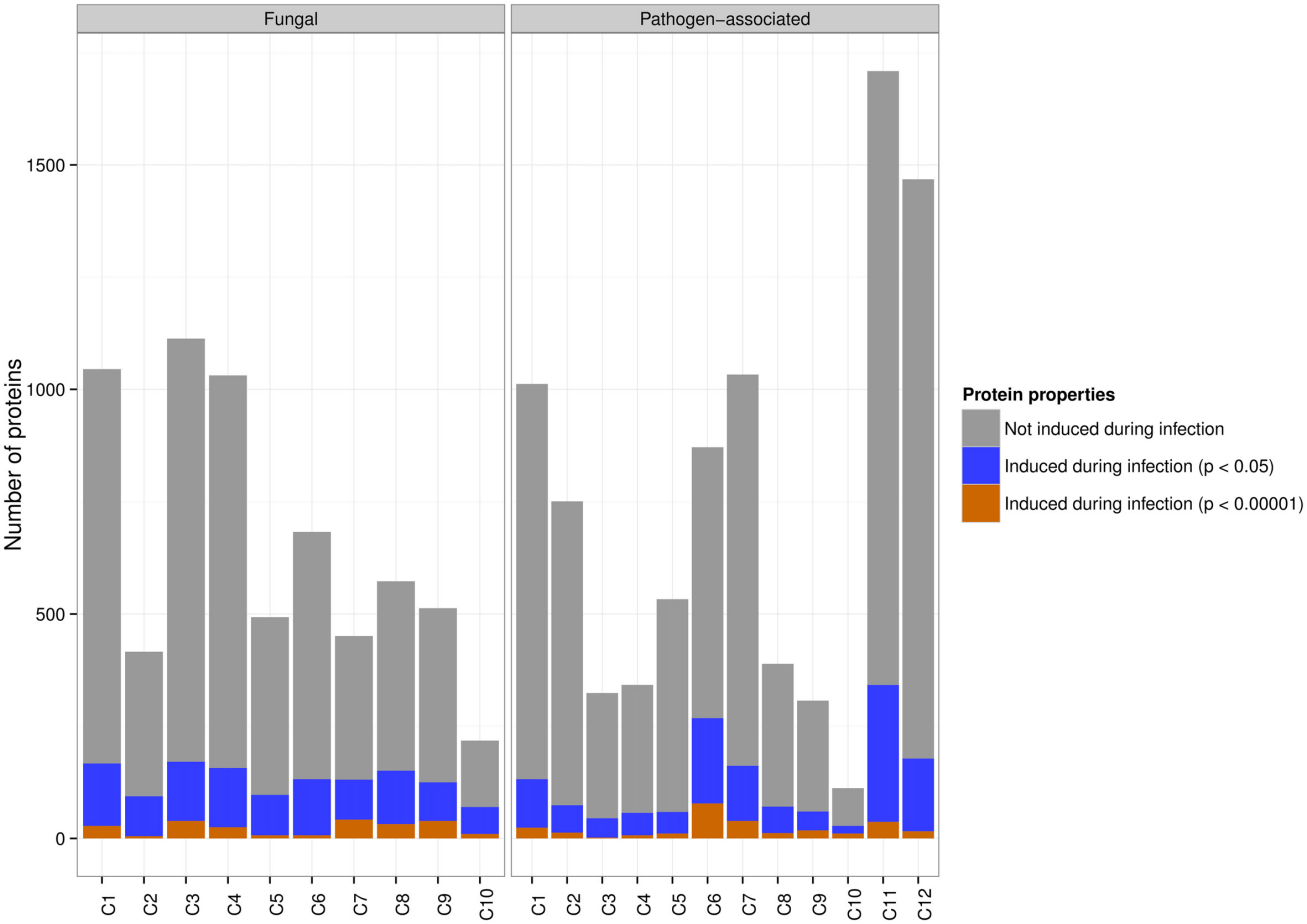
**Both fungal and pathogen-associated wheat stem rust gene families are *in planta* induced**. The majority of pathogen-associated and fungal genes are not up-regulated during infection. At a significance threshold of *p* < 0.05, 19.8% of genes from fungal gene families are up-regulated during infection and are distributed across all clusters. 16.6% of genes from pathogen-associated gene families are up-regulated during infection. At a significance threshold of *p* < 0.00001, the highest number of genes up-regulated during infection is found in the pathogen-associated cluster C6 (secreted, cysteine-rich).

**Figure 3:**
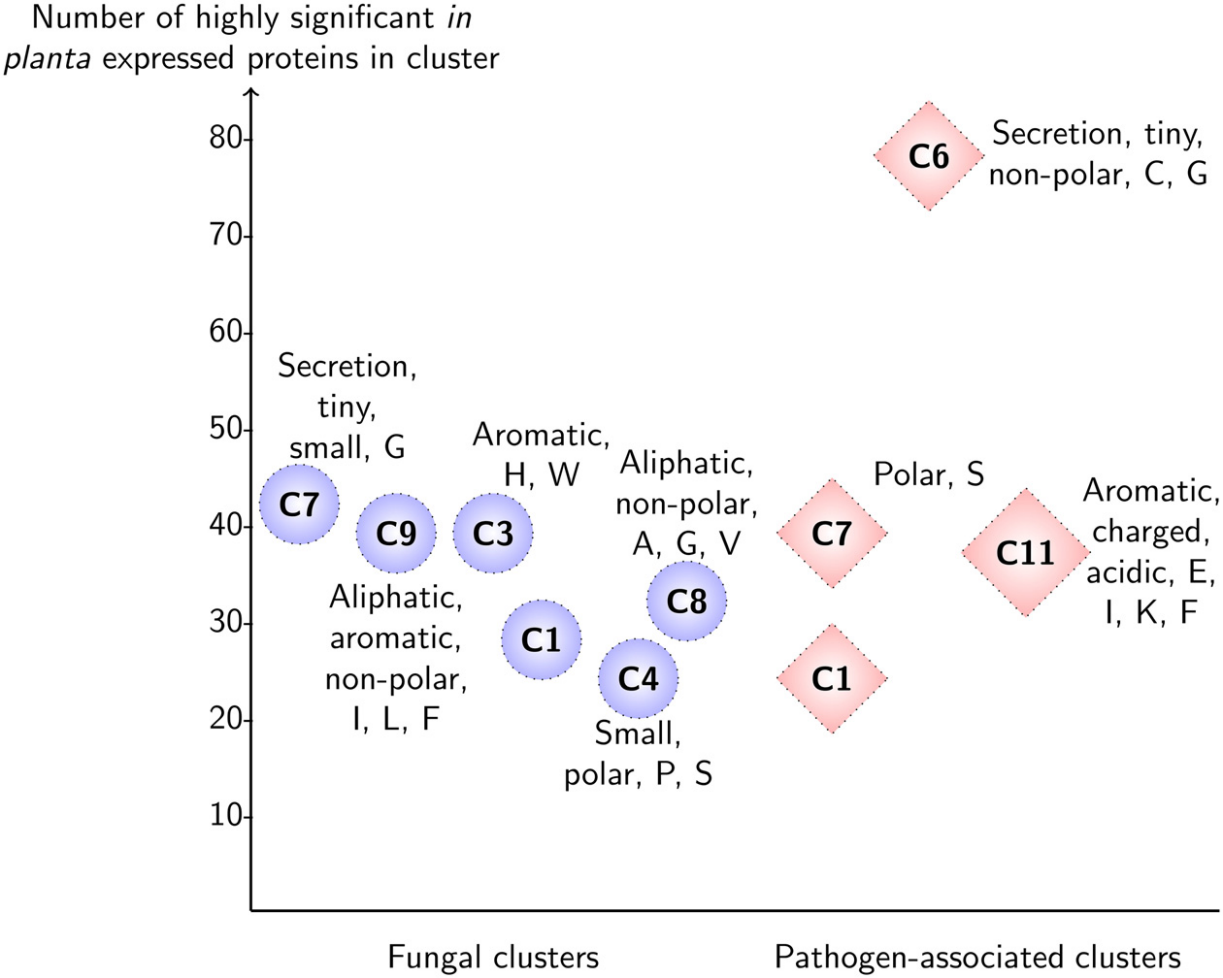
**Highly significant *in planta* expression for predicted clusters of the fungal gene families and pathogen-associated gene families in wheat stem rust**. For each cluster, the enriched sequence-derived features and number of highly significant up-regulated genes are shown (*p* < 0.00001). Note that for clarity, only clusters which have more than 20 up-regulated proteins are shown. The pathogen-associated cluster C6 sits at the top with 78 proteins that are up-regulated with high significance and has enrichment in features that are associated with effector proteins (secreted, small size, cysteine-rich).

At significance threshold *p* < 0.05, up-regulation was found for 1476 of 8851 (16.6%) genes in the pathogen-associated gene family clusters, whereas up-regulation was detected for 1295 of 6536 (19.8%) genes in the fungal gene family clusters (Figure 2). A striking difference in the distribution of up-regulated stem rust genes with high significance (*p* < 0.00001) can be observed across the clusters (Figure 3). The pathogen-associated, secreted, cysteine-rich cluster C6 contains the highest number of genes that are up-regulated. However, we do observe highly significant up-regulation during infection also across the fungal gene families. This can be expected as the stem rust activates a diverse number of proteins during infection and not all of these will act as an effector or have a function directly related to pathogenesis. For example, haustoria play a major role in nutrient uptake from the host during infection and show high expression of sugar and amino acid transporters (Garnica et al., 2013). Furthermore, not all fungal effector proteins can be expected to be part of the small, cysteine-rich cluster C6. Additional pathogen-associated clusters show potential of containing effector candidates, with elevated levels of highly significant up-regulation during infection in clusters C1, C7, and C11 (Table 1, Figure 3). 9.7, 4.7, and 14.4% of proteins in clusters C1, C7, and C11, respectively, are predicted to be secreted by SignalP 4.1 and could thus contain putative effector candidates. Therefore, signatures of diversifying selection are used in the following as an additional line of evidence for pathogenicity function.

### *In planta* induced genes undergoing diversifying selection are predominantly found amongst pathogen-associated gene families

Because of the potential and pressure for co-evolution with the host, fungal pathogen effectors and avirulence genes may undergo accelerated rates of diversification and are thus likely to show signs of site-specific diversifying selection. In order to have sufficient evolutionary divergence, diversifying selection analysis was performed for the publicly available genomes of the Pucciniomycotina branch of the fungal kingdom (see Materials and Methods). In total, 10,213 stem rust genes with at least two orthologs across the Pucciniomycotina branch of the fungal kingdom were analyzed for signatures of site-specific diversifying selection. 4752 of these genes belong to the pathogen-associated gene families, 5177 belong to the fungal gene families and 284 genes are not part of gene families. In total, 387 of the 10,213 genes (3.8%) were found to show site-specific diversifying selection using two likelihood ratio tests of CODEML in the PAML software. Despite the higher number of genes in the fungal gene families, site-specific diversifying selection was predominantly detected across pathogen-associated gene families (Table 3). In total, 341 of the 387 (88.1%) rapidly evolving genes are part of pathogen-associated gene families, whereas only 43 are part of fungal gene families.

**Table 3:** **Diversifying selection in the wheat stem rust is predominantly detected across pathogen-associated gene families**.

	**Additional criteria**	**# of genes**	**# of genes under diversifying selection**
*P. graminis* f. sp. *tritici* genes with at least two orthologs	–	10,213	387 (3.8%)
	Member of pathogen-associated gene family	4752	**341 (7.2%)**
	Member of fungal gene family	5177	43 (0.8%)
	Not a member of gene family	284	3 (1.1%)

*10,213 P. graminis f. sp. tritici genes with at least two orthologs were analyzed for site-specific diversifying selection using two likelihood ratio tests of CODEML. The majority of rapidly evolving genes are part of pathogen-associated gene families*.

**Figure 4:**
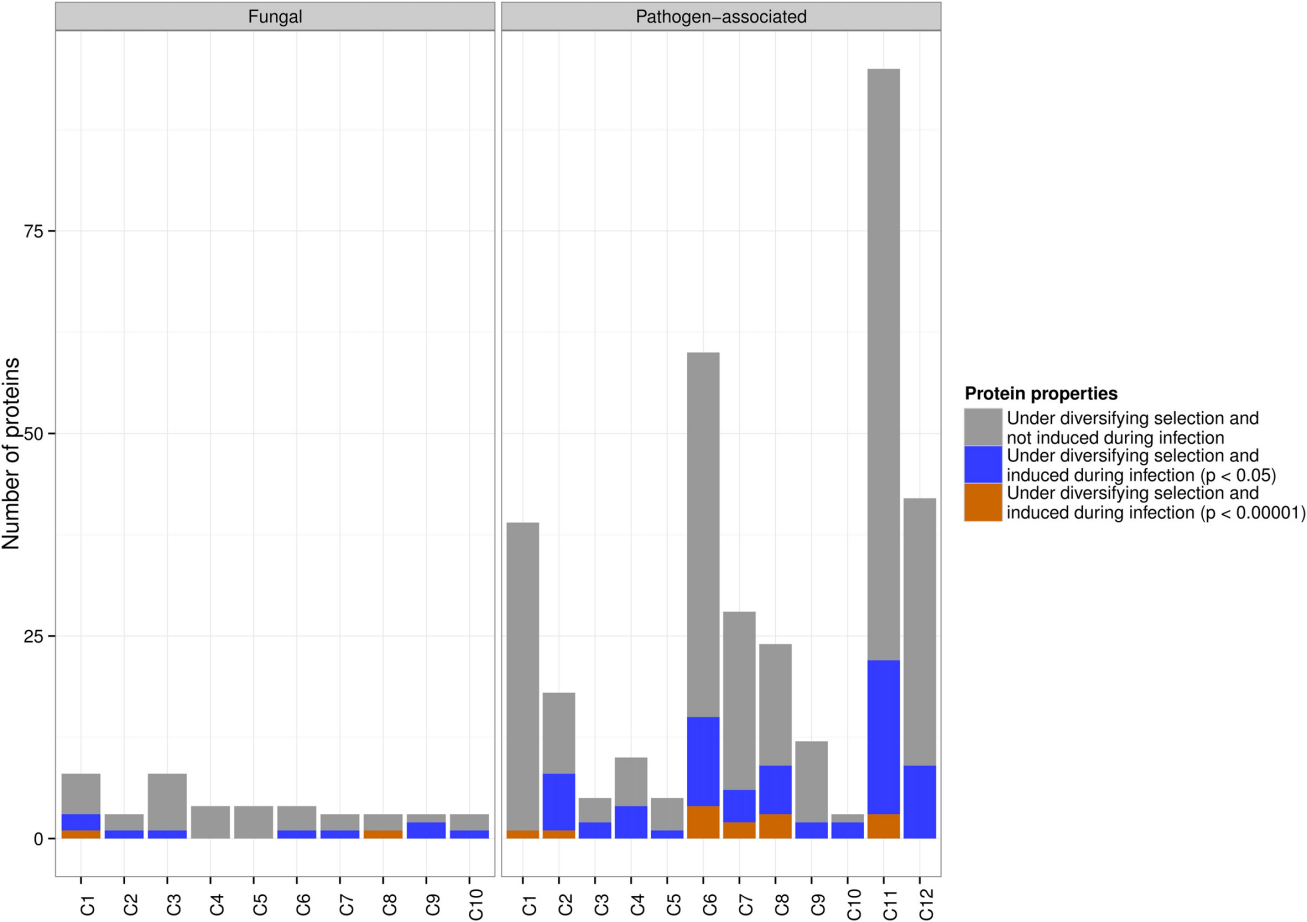
**The distribution of pathogen-associated and fungal genes with *in planta* up-regulation and diversifying selection across the clusters**. Diversifying selection is predominantly predicted for genes that are part of pathogen-associated gene families. At a significance threshold of *p* < 0.05, 81 pathogen-associated genes are rapidly evolving, compared to only 11 fungal genes. At a significance threshold of *p* < 0.00001, 14 rapidly evolving genes are predicted across the pathogen-associated gene families, with the majority of these being part of clusters C6, C7, C8 and C11.

Despite the fairly similar distribution of *in planta* up-regulated genes across the pathogen-associated and fungal gene families, site-specific diversifying selection using PAML was almost exclusively detected across pathogen-associated gene families. In particular, 81 up-regulated genes (*p* < 0.05) from pathogen-associated gene families were predicted to undergo diversifying selection whereas only 11 up-regulated genes from fungal gene families are rapidly evolving. A more stringent significance threshold of *p* < 0.00001 returned 14 genes from pathogen-associated gene families and two genes from fungal gene families that are undergoing diversifying selection (Figure 4).

Only one of the up-regulated genes (*p* < 0.05) in the fungal secreted cluster C7 is predicted to undergo site-specific diversifying selection, which indicates that these secreted proteins might have roles that do not require adaptation to the host or the environment. In contrast, the pathogen-associated secreted, cysteine-rich cluster C6 contains 15 up-regulated genes that are predicted to be undergoing diversifying selection. However, other pathogen-associated clusters such as C11 also contain a high number of rapidly evolving genes that are up-regulated during infection (Figure 4), which underlines the need to look beyond secreted, small and cysteine-rich as criteria for effector candidates.

Taken together, these results suggest that diversifying selection in *P. graminis* f. sp. *tritici* occurs predominantly across gene families that can be associated with pathogenicity via comparative genomics. However, signatures of diversifying selection cannot exclusively be linked to host adaptation or a role in pathogenicity. Therefore, a combination of pathogen-association, signatures of diversifying selection and expression data will be used to identify a small set of highly likely effector candidates.

### Pathogen-association, rapid evolution and up-regulation during *in planta* infection and in haustoria defines prime effector candidates

Prime fungal effector candidates can be expected to be highly expressed during infection and to be experiencing diversifying selection, reflecting the evolutionary arms race between host and pathogen. When searching for signatures of adaptive evolution in proteins that are highly induced during infection, we found site-specific diversifying selection almost exclusively in those that are pathogen-associated. To further prioritize the effector candidates, we used haustorial expression data of germinated urediniospores versus isolated haustoria of wheat stem rust strain 21-0 (RNAseq, Upadhyaya et al., in press). Forty six genes were found to be under diversifying selection, up-regulated during *in planta* infection and to have a fold change >2 when comparing expression in haustoria to germinated urediniospores (Table 4). Forty two of these 46 candidates are part of pathogen-associated gene families and are thus strong candidates for having a role in pathogenicity.

**Table 4:** ***P. graminis* f. sp. *tritici* genes that are predicted to undergo site-specific diversifying selection with significant *in planta* up-regulation (wheat infection to germinated urediniospores, *p* < 0.05, fold change > 2) and a fold change > 2 when comparing isolated haustoria to germinated urediniospores**.

**Pathogen-associated cluster**	**Protein**	**Secretion signal**	**aas**	**Cys**	**Rust orthologs**	**PFAM domain**	**Wheat/Spores (FC)**	**Haustoria/Spores (FC)**
C11	PGTG_08638	Yes	292	1	Stripe, leaf	–	187.1	1085.9
Fungal	PGTG_16225	Yes	310	7	Stripe, leaf	–	43.8	239
C11	PGTG_04972	–	340	–	Stripe, leaf	–	21.8	147.7
No gene family	PGTG_14091	Yes	502	1	Stripe, leaf	–	25.8	145.5
C12	PGTG_09276	–	134	–	Stripe	–	4.7	142.4
C6	PGTG_05592	Yes	257	10	Stripe	–	48.2	118.7
C1	PGTG_05174	–	239	1	Stripe, leaf, flax	–	32.6	81.2
C6	PGTG_11727	Yes	336	11	Stripe	–	9	56
C2	PGTG_06244	Yes	277	1	Leaf	–	4.2	53.7
Fungal	PGTG_17076	–	67	–	Stripe, leaf	Seed maturation protein (PF04927, 2.9e-07)	118.8	50.8
C6	PGTG_03859	Yes	165	11	Stripe, leaf	–	19.8	44.8
C11	PGTG_16303	Yes	441	4	Stripe	–	3.1	31.6
C8	PGTG_10538	Yes	411	–	Stripe, leaf	–	31.9	31.6
C8	PGTG_10539	Yes	392	2	Leaf	–	13.9	30.1
C10	PGTG_09318	Yes	87	8	Stripe, leaf	–	77	19.2
C11	PGTG_00341	–	361	2	Stripe, leaf	–	4.9	16.6
C2	PGTG_10398	–	1011	3	Stripe, leaf	–	22.8	13.5
C11	PGTG_14389	–	298	1	Stripe	–	3.9	11.4
C6	PGTG_17308	Yes	125	7	Stripe	–	5.9	8.3
C4	PGTG_01642	–	1329	2	Stripe, leaf	–	2.7	7.3
C11	PGTG_10056	Yes	766	12	Stripe, leaf	–	37.1	7.3
C7	PGTG_03213	–	565	–	Stripe, leaf	–	25.6	7.2
C11	PGTG_15791	–	376	4	Stripe	–	2.7	7.2
C8	PGTG_13414	Yes	197	2	Stripe, leaf	–	45.8	6.6
C4	PGTG_01631	Yes	807	5	Stripe, leaf	–	7.5	5.6
C11	PGTG_15481	Yes	502	3	Stripe, leaf	–	2.3	5.6
C11	PGTG_17733	Yes	482	–	Leaf	–	9.5	5.3
C8	PGTG_10625	Yes	474	3	Stripe	–	5	5.3
C11	PGTG_18622	–	300	2	Stripe, leaf	–	2.5	4.9
C2	PGTG_07786	–	854	3	Stripe, leaf	–	2.6	4.4
C11	PGTG_12173	–	629	4	Stripe, leaf	–	6.1	3.9
C2	PGTG_07911	Yes	324	4	Stripe, leaf	–	7	3.9
C6	PGTG_16750	Yes	124	8	Leaf	–	4.1	3.8
C6	PGTG_04109	Yes	100	9	Leaf	–	5.1	3.8
C6	PGTG_05119	Yes	141	6	Stripe, leaf	–	61.3	3.7
Fungal	PGTG_05197	–	537	9	Leaf	Sugar (and other) transporter (PF00083, 3e-76)	5.7	3.7
C7	PGTG_07078	–	324	–	Stripe, leaf, flax	Ribosomal protein S10p/S20e (PF00338, 1.2e-16)	2.6	3.2
C12	PGTG_17001	–	878	13	Stripe, leaf	–	2.6	3
C11	PGTG_15702	–	334	15	Stripe, leaf, flax, poplar	–	2.3	2.8
C3	PGTG_06359	–	734	13	Stripe, leaf	–	6.2	2.7
C6	PGTG_17534	Yes	114	4	Stripe	–	18.1	2.4
C11	PGTG_14388	Yes	353	1	Leaf	–	2.5	2.2
C9	PGTG_19205	–	299	5	Leaf	–	9.7	2.1
C9	PGTG_14673	Yes	189	10	Stripe	–	6.4	2.1
C11	PGTG_15389	–	667	2	Leaf	–	2.2	2.1
C8	PGTG_10832	–	149	3	Leaf	–	2	2
C12	PGTG_17927	–	711	9	Stripe	Alpha-kinase family (PF02816,1.4e-32)	5.5	2

*For each protein, its signal peptide prediction by Signal P 4.1, protein sequence length (aas), number of cysteines (Cys) and distribution of wheat stem rust orthologs across the Pucciniomycotina genomes are given. Stripe stands for P. striiformis PST-78, leaf for P. triticina 1-1 BBBD Race 1, flax for M. lini and poplar for M. laricis-populina. Note that no proteins undergoing diversifying selection were found in pathogen-associated cluster C5. Protein expression levels are given for the two experiments (FC: fold change). The genes are ordered by decreasing haustorial differential expression fold change*.

A common feature of fungal effectors is that they currently lack functional annotation. Using the motif search tool MEME (Bailey et al., 2009), we could not detect a common sequence motif for the 46 proteins under diversifying selection. Only four of the 46 genes could be functionally annotated using Pfam searches. PGTG_17076 has a Pfam hit to seed maturation proteins (PF04927), PGTG_05197 to sugar transporter proteins (PF00083), PGTG_07078 to ribosomal proteins (PF00338) and PGTG_17927 to the alpha-kinase family (PF02816). Only three of the 46 proteins under diversifying selection have orthologies outside the concentrated group of *P. graminis* f. sp. *tritici, P. striiformis*, and *P. triticina*. The remaining proteins under diversifying selection share orthology with the wheat pathogens stripe rust and leaf rust, but not with other rust fungi. Similarly, all of the known rust Avr proteins in *M. lini* occur in families that are restricted to this species or its relative *M. larici-populina* and are not shared across other rust genera (Nemri et al., 2014). Thus, the observed genus level specificity of these candidates is consistent with their suspected roles in host-pathogen interactions.

**Figure 5:**
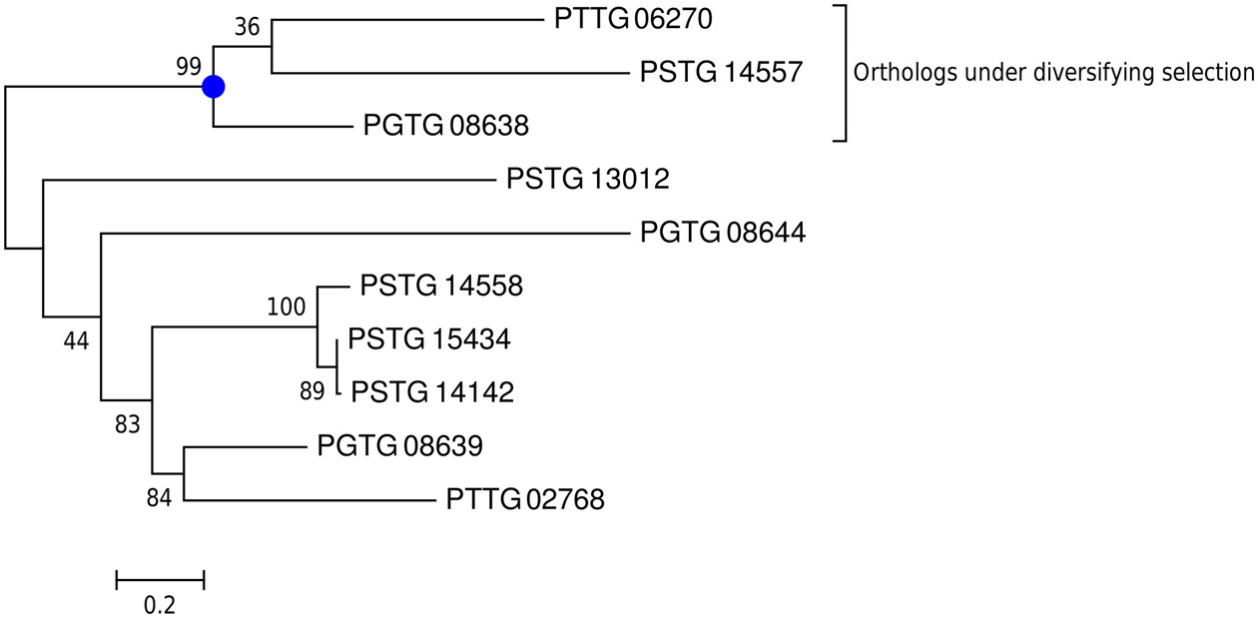
**For the gene family of protein PGTG_08638, diversifying selection is only detected for the orthologs**. A phylogenetic tree for the gene family of PGTG_08638 was predicted using PhyML and branch support values are shown. PAML detects site-specific diversifying selection only for the branch with the three orthologs (stem rust PGTG 08638, stripe rust PSTG_14557, leaf rust PTTG 06270), but not for the other variants PGTG_08639 and PGTG_08644 and their orthologs.

In particular, PGTG_08638, PGTG_16225, PGTG_04972, PGTG_14091, PGTG_09276, and PGTG_05592 are highly induced in haustoria (fold change > 100, Table 4). Indeed, one variant of protein PGTG_08638 was identified in a functional screen of candidate effectors from the stem rust fungus *P. graminis* f. sp. *tritici* as an inducer of host genotype-specific hypersensitive cell death in wheat (Upadhyaya et al., 2013). This is in agreement with our PAML analysis, which detects diversifying selection only for the three orthologs (stem rust PGTG_08638, stripe rust PSTG_14557, leaf rust PTTG 06270), but not for the other variants (Figure 5). From this data, one could speculate that a gene duplication event with successive diversification has led to a gain of function in the stem rust variant PGTG_08638. This protein is recognized inside wheat cells (Upadhyaya et al., 2013) and would therefore belong to a class of effectors that are delivered into host cells from haustoria. The other haustorially expressed gene candidates described here are likely also to be in this class.

By combining comparative information, expression data and signatures of diversifying selection, we have dramatically reduced the number of effector candidates reported in earlier studies (Duplessis et al., 2011; Saunders et al., 2012) to a small set of strong effector candidates. The computational prediction of an effector candidate in this set (PGTG_08638) that has been shown to induce host genotype-specific hypersensitive cell death in wheat (Upadhyaya et al., 2013) emphasizes that this set of 46 effector candidates could be the basis of a priority list for laboratory verification. Furthermore, protein PGTG_08638 is likely to have been missed by effector prediction pipelines that select candidates based on a secretion signal, small size and high number of cysteines, as it only contains one cysteine residue in its sequence of 292 amino acids. Indeed, in the work by Saunders et al. (2012), PGTG_08638 was predicted to be in a tribe that was ranked below the cut-off score and was thus dismissed as an effector candidate. Our results also confirm the presence of adaptive diversification of wheat stem rust gene families involved in pathogenicity and will lead to further insight into how this successful pathogen overcomes host resistance.

## Discussion

Understanding how filamentous fungal plant pathogens cause disease is of utmost importance due to the devastating losses they cause in important crop plants. In particular, the wheat stem rust *Puccinia graminis* f. sp. *tritici* has demonstrated the ability to rapidly overcome resistant host lines and is currently threatening the majority of the global wheat cultivars (Singh et al., 2011). The prediction of genes involved in host-pathogen interactions, in particular effector proteins that interact directly with target host defense proteins, has thus far been difficult for fungal pathogens due to a lack of signature sequence motifs. Previous efforts to understand virulence largely relied on the prediction of secreted, small and cysteine-rich proteins as candidate effectors (Ellis et al., 2009), which is likely to return an overwhelming number of candidates for experimental verification. Furthermore, there is growing evidence that effectors can have unconventional characteristics, such as no predicted signal peptide, a low number of cysteine residues or a large size (Sperschneider et al., 2013).

In this work, we introduced an alternative bioinformatics pipeline that prioritizes effector candidates by combining multiple lines of evidence such as taxonomic information, expression data and evolutionary signatures of diversifying selection. This approach was applied to the wheat stem rust *P. graminis* f. sp. *tritici*, which features lineage-specific expanded gene families that are thought to drive genome innovation, adaptation and pathogenicity-associated processes (Duplessis et al., 2011; Raffaele and Kamoun, 2012). Unsupervised clustering of the predicted gene families based on sequence-derived protein features revealed that pathogen-associated and fungal gene families form distinct clusters and that elevated levels of up-regulation during infection can be found across both classes. Our observation is that expression data is likely to be the line of evidence with the greatest detection power but probably the least discriminatory as a large number of genes can be expected to be up-regulated during infection that do not necessarily correspond to effectors. The ability of expression data to capture effectors highly depends on choosing the right time points and on the power of the experimental setup to mimic infection under field conditions. In pathogens where haustorial tissue can be extracted such as the wheat stem rust fungus, expression data is likely to be a powerful line of evidence. In other pathogens there can be issues with capturing early time points for effector expression and thus, the benefits of using expression data may be of lesser value. In contrast, *in planta* induced genes undergoing diversifying selection are predominantly found amongst pathogen-associated gene families. This indicates that pathogen-associated gene families in *P. graminis* f. sp. *tritici* might be involved in host adaptation or pathogen specialization. The diversifying selection analysis is likely to be highly discriminatory, but may miss effector candidates where the selection signal is not strong. This approach clearly becomes more powerful the more species are available for a certain lineage.

In particular, this analysis revealed a set of 42 effector candidates in *P. graminis* f. sp. *tritici* that are part of pathogen-associated gene families, up-regulated during infection and at the same time rapidly evolving in a suspected evolutionary arms race with the host (Table 4). Furthermore, we find that the majority of these effector candidates are part of pathogen-associated clusters that lack enrichment in features commonly associated with fungal effectors such as small size and high number of cysteines. Recently, one of our predicted effector candidates was shown to have a variant that induces host genotype-specific hypersensitive cell death in wheat in a functional screen of candidate effectors from the stem rust fungus *P. graminis* f. sp. *tritici* (Upadhyaya et al., 2013). Therefore, we are confident that this small set of effector candidates should be the future target for laboratory experiments.

We propose that there is a need to expand the criteria for predicting effectors beyond secreted, small and cysteine-rich. Instead, a combination of the three signals (taxonomic information, expression data and diversifying selection) can be a powerful and unbiased predictor for genes involved in host-pathogen interactions. Our diversifying selection pipeline can be applied to a wide range of pathogens for which divergence data is available and can give insight into the evolutionary processes between host and pathogen. In particular, our work indicates that elements of the genome linked to pathogenicity are evolutionarily dynamic, possibly in mechanisms relating to host adaptation or pathogen specialization. Therefore, effector candidates in plant pathogens that show signs of rapid evolution are promising targets for disease control in crop plants.

### Conflict of interest statement

The authors declare that the research was conducted in the absence of any commercial or financial relationships that could be construed as a potential conflict of interest.

## Supplementary material

Table S1 **The list of fungal genomes used for comparative classification of gene families into pathogen-associated and fungal**.
